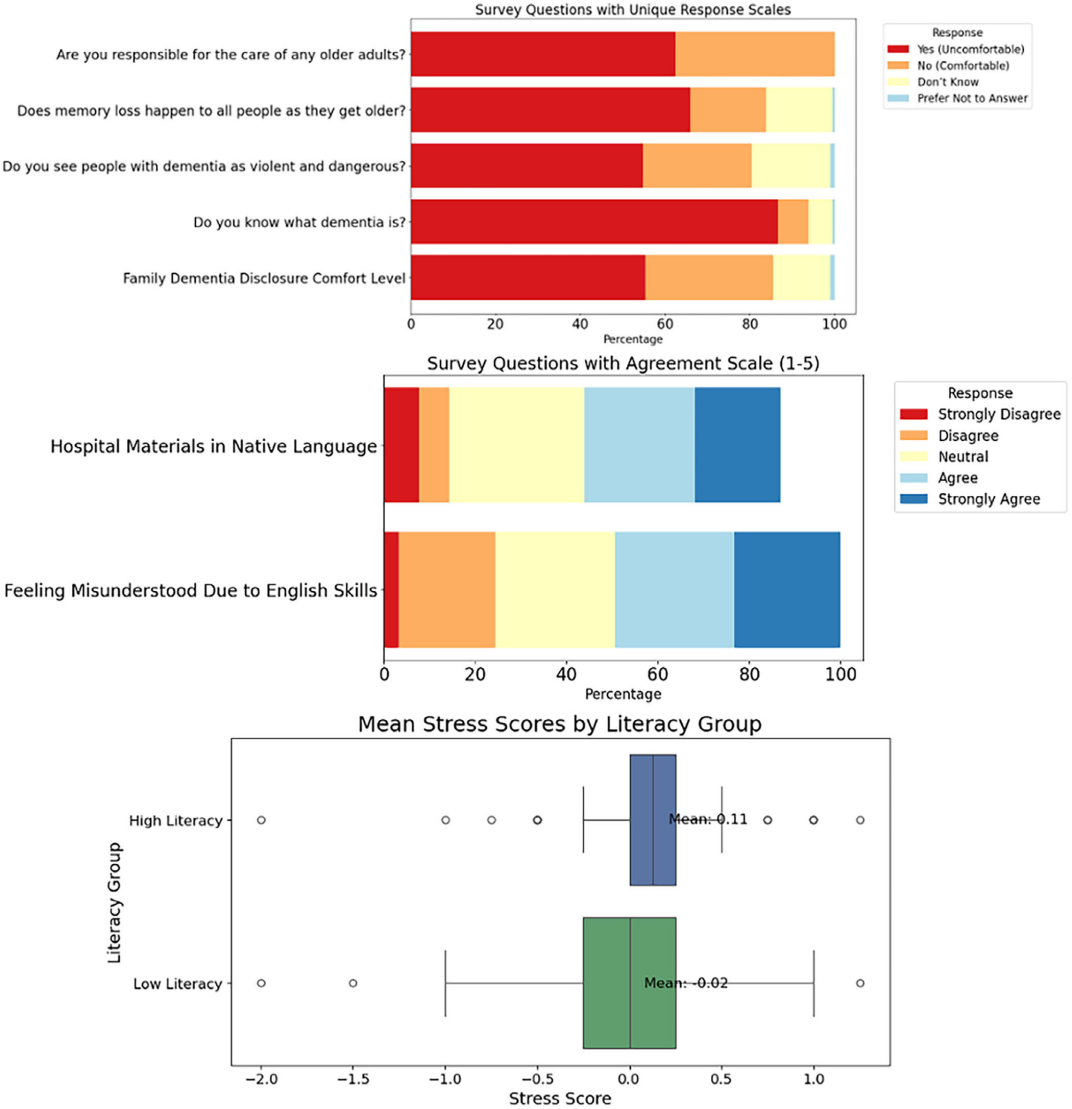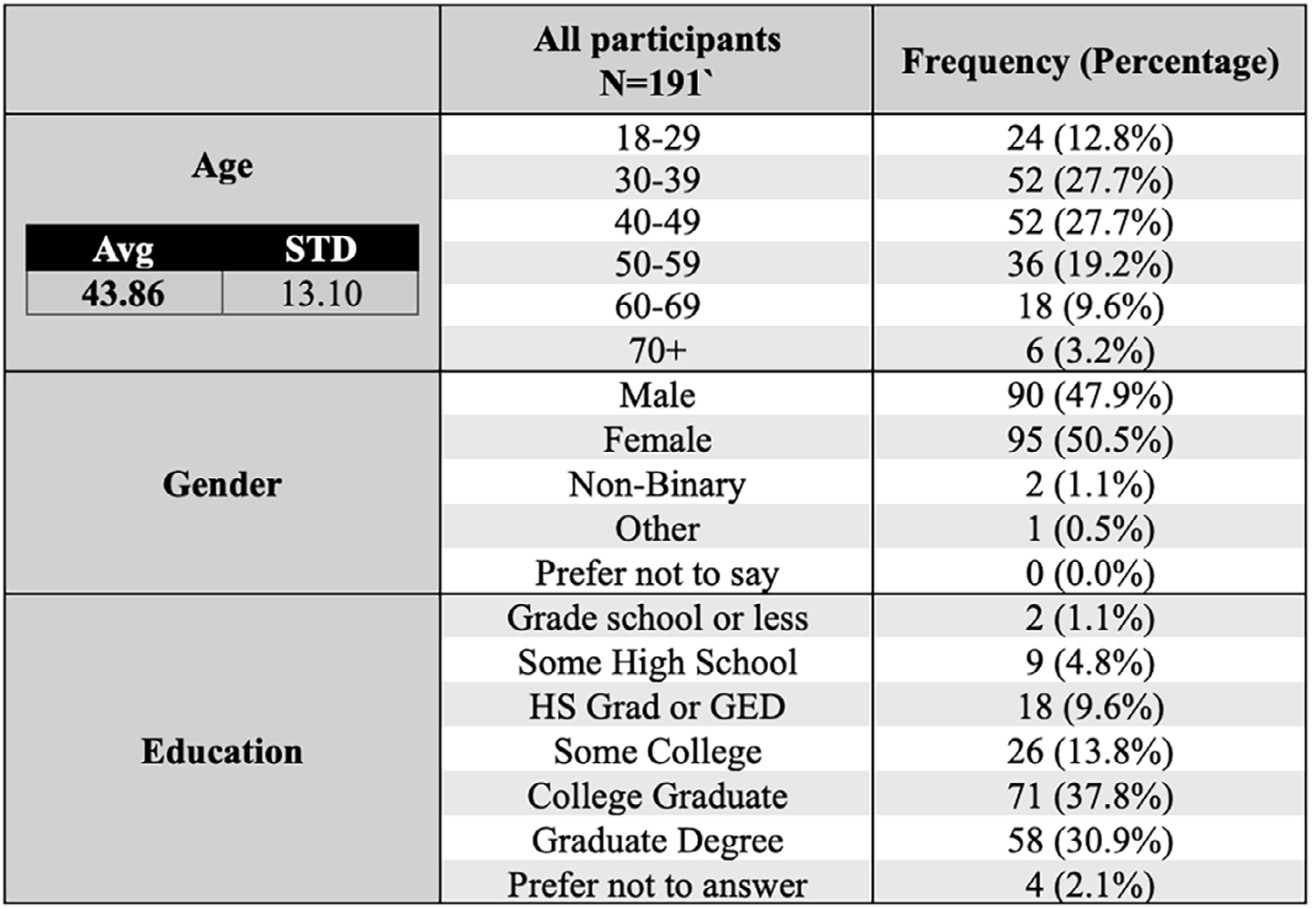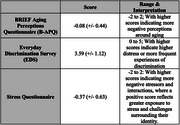# Understanding aging and dementia among South Asians in the Greater Atlanta region

**DOI:** 10.1002/alz70858_102442

**Published:** 2025-12-24

**Authors:** Yash Kamdar, Mansura Khanam, Adil Muhammad, Neelum T. Aggarwal, Ambar Kulshreshtha

**Affiliations:** ^1^ Emory University, Atlanta, GA, USA; ^2^ Rush Alzheimer's Disease Center, Chicago, IL, USA; ^3^ Rush University Systems for Health, Chicago, IL, USA; ^4^ Rush University Medical Center, Chicago, IL, USA; ^5^ Emory University School of Medicine, Atlanta, GA, USA

## Abstract

**Background:**

More than 5 million South Asians from India, Pakistan, Bangladesh, Nepal, Sri Lanka, and Bhutan live in the United States, and it is one of the fastest‐growing populations. South Asians have a disproportionately high burden of type 2 diabetes and have double the risk of cardiovascular disease compared to other ethnic groups. Understanding current attitudes about health, aging, and dementia will provide important perspectives in developing health programs tailored for South Asians. The South Asian Healthy Aging Research project aims to assess attitudes toward health and aging and evaluate the role of social factors. The goal is to develop a national registry for culturally tailored education programs for this population subgroup.

**Methods:**

A cross‐sectional study was conducted in 2023‐24 where participants from the Greater Atlanta area who self‐identified as South Asian completed validated questionnaires. These included the BRIEF Health Literacy Screening Tool, BRIEF Aging Perceptions Questionnaire, Everyday Discrimination Scale, and Perceived Stress Scale. Participants were recruited through community outreach efforts in Greater Atlanta. Data analysis was performed using RStudio.

**Results:**

191 participants completed the surveys, with an average age of 43.9 years, and 50.5% were female. Approximately 80% were first‐generation immigrants, and 97% had an educational level of high school or above. Despite the education levels, 10% agreed or strongly agreed with hesitating to seek medical help in the U.S. In addition to reporting high levels of stress and experiencing discrimination (25%), language barriers were a concern, with 23% feeling misunderstood due to their language and 41% preferring healthcare materials in their native language. More than 64% were aware of someone living with dementia but had misconceptions with 66% believing memory loss is inevitable with age. 30% reported stigma with the condition, and there was a general lack of knowledge of dementia risk factors.

**Conclusions:**

Our results demonstrate a lack of awareness about aging and dementia and high levels of chronic stress in this group of South Asians. The findings highlight the necessity for culturally tailored outreach and educational programs to improve awareness of healthy aging and brain health in this high‐risk population.